# Functional Ingredients from Food Waste and By-Products: Processing Technologies, Functional Characteristics and Value-Added Applications

**DOI:** 10.3390/foods14050847

**Published:** 2025-02-28

**Authors:** Lucía Seguí, Cristina Barrera

**Affiliations:** Instituto Universitario de Ingeniería de Alimentos—FoodUPV, Universitat Politècnica de València, Camino de Vera, s/n, 46022 Valencia, Spain; mcbarpu@tal.upv.es

## 1. Introduction

In the current scenario, food waste stands out as a pressing issue, accounting for a significant portion of the waste generated worldwide [1]. It arises throughout the food chain, from production to overharvesting, processing, transportation, storage, and consumption [2]. Every year, about 1.3 billion tons of edible food are wasted worldwide, of which 60 million tons are produced in the European Union [2,3]. Despite over half of food waste being generated at the consumption stage, postharvest and processing losses also account for an important share. Reducing food loss and waste is a key challenge that must be addressed to achieve the Sustainable Development Goals defined by the United Nations in the 2030 Agenda [4].

Tackling food waste presents advantages for the climate, for food security and for agri-food system sustainability [3]. In order to meet current food industry sustainability and resource efficiency needs, valorizing food waste, by-products and surpluses has become both a challenge and a necessity. Often rich in nutrients and bioactive compounds, food waste and by-products hold immense potential to be transformed into valuable functional ingredients with applications in the food industry and other related areas [5,6,7]. This Special Issue aimed to explore processing technologies for obtaining functional ingredients from these underutilized resources, and to expand knowledge about the nutritional, technological and functional characteristics of the new ingredients and value-added products that can be obtained, exploring their wide-ranging applications across different sectors, from food and beverages to agriculture, packaging and beyond.

As a result, this Special Issue gathers eleven valuable scientific contributions in the form of original research works which could be categorized in two main groups: one dealing with processing and extraction techniques to obtain value-added products or ingredients, and another focused on new product development and other applications.

## 2. Article Overview

In this SI, various technologies are investigated to produce value-added products or ingredients from food waste and by-products. Maté et al. (Contribution 1) explore subcritical water extraction and hydrogen peroxide alkaline delignification to fractionate grape stalks, a lignocellulosic biomass from the winemaking process. Subcritical water extraction allowed for obtaining phenolic-rich extracts with more than 70% of the total phenolic content of the grape stalks, whereas a part of these compounds also remained in the solid fraction. After subcritical water extraction, the products displayed improved their polyphenolic contents and antioxidant activities compared to the original material. The solid residue fraction was subjected to a green bleaching process to purify cellulose. The first bleaching cycle led to a maximum reduction in the lignin content of the insoluble fractions of 75%, with a higher cellulose purity in the samples obtained at 170 °C and bleached with 4% alkaline hydrogen peroxide. The final lignin content in the bleached samples was still higher than 25%, thus further studies are proposed to optimize cellulose recovery.

In another paper, Bas-Bellver et al. (Contribution 2) propose a series of thermophysical and biological treatments to improve the antioxidant properties of broccoli stem products to eventually produce functional powdered ingredients. Ultrasounds, microwaves, autoclaving, pasteurization and lactic acid fermentation are applied as pretreatments to enhance the antioxidant properties of the ground broccoli residues, and the corresponding powders obtained after air- or freeze-drying. The pretreatments applied enhanced the antioxidant properties of broccoli wastes, particularly ultrasounds and pasteurization treatments, followed by fermentation and, finally, autoclaving and microwaving. Microscopic observations suggested that membrane permeabilization and cell breakage were related to this improvement. When applied to non-pretreated samples, drying improved the antioxidant properties of the flours; this effect was more significant in air-dried samples, since higher temperatures promote the formation of certain antioxidant compounds. However, combining the proposed pretreatments with dehydration was not successful in improving antioxidant properties, except for the ultrasonicated samples. It is concluded that pretreatments may be applied to enhance the antioxidant attributes of broccoli waste, but extending this to dried products was not demonstrated and requires further research.

Other than as a pre-treatment, ultrasounds are proposed to assist the extraction of different constituents from food wastes. On the one hand, Zhang et al. (Contribution 3) proposed ultrasounds for extracting proteins from *Dictyophora rubrovolvata* volva and modifying their techno-functional properties. A maximum 43% extraction rate could be achieved in the ultrasound-assisted process. Compared to conventional alkaline extraction, ultrasonication had an impact on proteins’ morphology and molecular size; the secondary and tertiary structures of the extracted proteins were modified. Changes in the proteins’ characteristics and inter-molecular forces led to significant improvements in techno-functional properties such as water and oil holding capacity, foaming capacity and stability, emulsion activity, and stability, thus leading to new applications for food product development. On the other hand, Leonarski et al. (Contribution 4) proposed an ultrasound-assisted extraction process to obtain an anthocyanin-rich extract from black rice bran. The obtained crude and resin-purified extracts were evaluated in terms of functional properties and biological activities by determining anthocyanin content, antioxidant activity, antidiabetic and antitumoral activities, cytotoxicity and oxidative stress. The process was successfully scaled up 20 times without negatively affecting anthocyanin recovery. The extracts exhibited antidiabetic and anticancer effects, showing promising potential. The authors suggest further research to verify whether the extracts can be proposed as alternative therapeutics to control hyperglycemia in diabetic patients or as a potential anticancer supplement.

Non-communicable diseases such as diabetes, cancer or obesity are closely related to human gut microbiota. Hence, improving gut microbiota diversity is critical for developing healthier diets. In this vein, Li et al. (Contribution 5) investigated the prebiotic activity of a novel polysaccharide extracted from Huangshui, a by-product of traditional Chinese Baijiu production. This polysaccharide was isolated by gradient ethanol precipitation and was found to mainly be composed of arabinose, xylose and glucose in a molar ratio which differed from those previously reported. The novel polysaccharide was used by gut microbiota during in vitro fecal fermentation and was found to regulate the composition and abundance of beneficial microorganisms such as Phascolarctobacterium, Parabacteroides, and Bacteroides. The prebiotic activity of the polysaccaride was also evidenced by the production of short chain fatty acids, including acetic, butyric, and propionic acid, during fermentation.

Postharvest losses also represent a waste of nutrients, and the resources used in their growth and harvesting. Addressing this, Hosseininejad et al. (Contribution 6) proposed the valorization of astringent ‘Rojo brillante’ persimmon through the development of energy bars formulated with dehydrated persimmons, walnuts, hazelnuts and chia seeds. The new formulated product showed higher levels of healthy fats, proteins, and fiber than other commercial energy bars. In vitro simulated digestion revealed a higher recovery index for soluble tannins than for carotenoids. The bars behaved satisfactorily during storage, since astringency was mitigated, and carotenoids and antioxidant activity remained stable. In this way, a discarded material was transformed into a sustainable nutritious option for the snack industry. Siczek et al. (Contribution 7) present another piece of research in which a by-product is used to formulate muesli bars. In this case, researchers aimed to evaluate the possibility of replacing walnuts with oil pomace in muesli bar recipes and assess whether the resulting product met quality standards. The pomace-enriched bars exhibited physicochemical properties comparable to those of control bars, with positive sensory evaluations. Walnut pomace is therefore presented as a sustainable ingredient, potentially expanding product diversity while reducing environmental impacts.

The beverage industry can also benefit from the valorization of food by-products. In this vein, sugarcane syrup, an industrial by-product, has potential for new product development in the fermented beverages sector. Lv et al. (Contribution 8) proposed the use of sugarcane syrup to partially replace barley in producing lager beer. Results evidenced that the fruit and hop aromas were more harmonious when 20% sugarcane juice was added to the basic wort. Under these conditions, the fermentation process was optimized. The beer brewed with sugarcane syrup met the basic specifications of beer, increasing the utilization of amino acids by the yeasts, and bringing new flavor compounds compared with the normal all-barley beer. It also showed greater resistance to aging. In regions where sugarcane is abundant, utilizing this versatile local raw material to replace barley in traditional brewing recipes offers the dual benefits of reduced production costs and more sustainable beer production.

Other than developing novel functional food products by the addition of upcycled ingredients, the value-added applications of food waste are vast. For instance, food by-products have potential as preservative agents due to their antioxidant and antimicrobial activities. In line with this, Martínez et al. (Contribution 9) presented a novel approach in which the preservative properties of melanin and melanin-free fractions of cephalopod ink were evaluated on canned golden seabream (*Sparus aurata*) fillets after 3 months of storage at room temperature. Samples treated with the ink at specific concentrations showed higher levels of free fatty acids and phospholipids compared to the control, although differences were not statistically significant. Other applications rely on improving food functionality by the indirect use of food wastes. For instance, Balanč et al. (Contribution 10) developed biodegradable nanofibers made from pumpkin leaf protein concentrate and gelatin through electrospinning. The nanofibers were designed as fast-dissolving systems for supplementing foods with nutraceuticals, specifically vitamin B12. The leaf protein concentrate not only facilitated a slower, sustained release of vitamin B12 over time, but also contributed to maintaining its thermal stability. A different approach is presented by Navajas-Porras et al. (Contribution 11), who investigated the use of chemically modified spent coffee grounds for the agronomic fortification of Dutch cucumbers. They focused on the effects on Zinc biofortification, antioxidant capacity and the production of short-chain fatty acids after in vitro digestion–fermentation. It is concluded that both the Zn content and chemical composition of cucumbers may vary significantly with growing conditions, thus determining their contribution to the dietary intake of nutrients and antioxidants.

## 3. Conclusions

In summary, this Special Issue “Functional Ingredients from Food Waste and By-Products: Processing Technologies, Functional Characteristics and Value-Added Applications” evidences the great interest in exploring new techniques for food waste and by-product valorization, as well as in developing appplications which contribute to produce healthier and more sustainable foods.
